# Supplementary material for: Every sip counts: Understanding hydration behaviors and user-acceptability of digital tools to promote adequate intake during early and late pregnancy
Source: PLOS Digit Health. 2024 May 7;3(5):e0000499. doi: 10.1371/journal.pdig.0000499 (PMC11075850; doi:10.1371/journal.pdig.0000499)
Supplement: S1 Questionnaire — (DOCX) [file pdig.0000499.s001.docx]

**DEMOGRAPHICS & HEALTH HISTORY**

Q1 Height (in)

________________________________________________________________

Q2 Pre-pregnancy Weight (in pounds)

________________________________________________________________

Q3 Current Weight (in pounds)

________________________________________________________________

Q4 Age (in years)

________________________________________________________________

Q5 What is your current blood pressure?  (Fillable field)

________________________________________________________________

Q6 What best describes your race/ethnic background?

- American Indian or Alaska Native (1)
- Asian (2)
- Native Hawaiian or Other Pacific Islander (3)
- Black or African-American (4)
- White (5)
- Two or more races (6)
- Other (please specify) (7) ________________________________________________

Q7 Are you of Hispanic, Latino, or of Spanish origin?

- Yes (1)
- No (2)

Q8 What is your current employment status?

- Employed, full-time (1)
- Employed, part-time (2)
- Retired (3)
- Student (4)
- Unemployed and looking for work (5)
- Unemployed and not looking for work (6)

Q9 What is your occupation?

________________________________________________________________

________________________________________________________________

Q10 What is your family income:

- < $10,000 (1)
- $10-20,000 (2)
- $20-40,000 (3)
- $40-100,000 (4)
- > $100,000 (5)
- Other (please specify) (6) ________________________________________________

Q11 What is the highest level of education you have completed?

- No schooling completed, less than 1 year, nursery, kindergarten, or elementary (1)
- Some high school (2)
- Completed high school or received GED (3)
- Trade or technical school certificate (4)
- Some college, no degree (5)
- Associate's degree (6)
- Bachelor's degree (7)
- Master's degree (8)
- Professional school degree (MD, JD, DDC) (9)
- Doctoral (PhD, EdD, ScD) (10)

Q12 Number of gestational weeks currently pregnant:

Q13 Estimated due date: _____ month _____ day _____ year

Q14 Are you experiencing/have you experienced any of these conditions during your pregnancy?

- Vaginal bleeding (1)
- Dizziness (2)
- Severe headaches or migraines (3)
- Calf pain or swelling (4)
- Decreased fetal movement (5)
- Fluid leakage (6)
- Severe cramping (7)
- Kidney stones or urinary tract infection (8)
- Impaired glucose tolerance (9)
- Anxiety (10)
- Depression (11)
- Hepatitis/liver disease (12)
- Blood transfusion (13)
- Digestive disorder/Irritable bowel syndrome (14)
- Hyperemesis gravidarum (15)
- Preeclampsia (16)
- Hypertension (17)
- Other (please explain): ___________________________________________

**HYDRATION QUESTIONS ADAPTED FROM KIDNEY STONES NEEDS ASSESSMENT AND sipIT QUESTIONNAIRE**

Q15 Do you know how much water you should be drinking in a day? (in ounces)

________________________________________________________________

Q16 Drinking water is something I do automatically.

- Strongly Disagree (1)
- Disagree (2)
- Somewhat disagree (3)
- Neither agree nor disagree (4)
- Somewhat agree (5)
- Agree (6)
- Strongly Agree (7)

Q17 Drinking water is something I do without having to consciously remember.

- Strongly Disagree (1)
- Disagree (2)
- Somewhat disagree (3)
- Neither agree nor disagree (4)
- Somewhat agree (5)
- Agree (6)
- Strongly Agree (7)

Q18 Drinking water is something I do without thinking.

- Strongly Disagree (1)
- Disagree (2)
- Somewhat disagree (3)
- Neither agree nor disagree (4)
- Somewhat agree (5)
- Agree (6)
- Strongly Agree (7)

Q19 Drinking water is something I start doing before I realize I’m doing it.

- Strongly Disagree (1)
- Disagree (2)
- Somewhat disagree (3)
- Neither agree nor disagree (4)
- Somewhat agree (5)
- Agree (6)
- Strongly Agree (7)

Q20 Do you keep track of your daily fluid consumption?

- Yes (1)
- No (2)
- Unsure (3)

Q21 Please describe the strategies you **CURRENTLY** use to track your fluid consumption:

________________________________________________________________

________________________________________________________________

________________________________________________________________

Q22 Have your drinking habits changed throughout pregnancy? For example, do you notice that you are drinking more or less as pregnancy progresses?

________________________________________________________________

________________________________________________________________

________________________________________________________________

Q23 Over the past week, how successful have you been at meeting fluid intake guidelines of at least 81 ounces/day (10 cups/day)?

- Not at all successful (0 days) (1)
- Rarely successful (1-2 days) (2)
- Sometimes successful (3-4 days) (3)
- Often successful (5-6 days) (4)
- Always successful (7 days) (5)

Q24 Which of the following have been barriers to meeting fluid intake guidelines of at least 80 ounces/day (10 cups/day)? Check all that apply.

- I am not thirsty enough (1)
- I forget to drink (2)
- It is a hassle to carry around a water bottle (3)
- I have to urinate too frequently if I drink that much (4)
- It’s hard to drink enough at work (5)
- I don’t like the taste of water (6)
- I am not aware of the need to drink more (7)
- It makes me feel bloated (8)
- Fluid is not easily available at work (9)
- I feel nauseous (10)
- It keeps me up at night (11)
- It is painful to drink that much with the baby pushing down on my bladder (12)
- I have a small bladder (13)
- I have acid reflux and it is uncomfortable (14)
- I have morning sickness (15)
- Other (specify): (16) ________________________________________________

Q25 What is your preferred beverage when you try to increase your fluid intake?

- Water (1)
- Flavored water (2)
- Tea/iced tea (3)
- Juice (4)
- Soda (5)
- Coffee (6)
- Other (7)

Q26 You selected other, please describe your preferred beverage when you try to increase your fluid intake.

________________________________________________________________

Q27 How frequently do you eat foods that are high in sodium, such as packaged soups or sauces,  fast food/take out, or salty snacks?  (Likert Scale of 1 time per week to >1 time per day)

- 1 time/week (1)
- 2 times/week (2)
- 3 times/week (3)
- 4 times/week (4)
- 6 times/week (5)
- 7 times/week (6)
- >1 time/day (7)

Q28 How frequently do you eat high protein foods, such as meat, fish, chicken, eggs, or protein supplements? (Likert Scale of 1-2 times per week to 3 or more times per day)

- 1-2 times/week (1)
- 3-4 times/week (2)
- 4-5 times/week (3)
- 6-7 times/week (4)
- 1-2 times/day (5)
- 3 or more times/day (6)

Q29 How frequently do you consume foods that are high in water, like fruits and vegetables or soup? (Likert Scale of 1-2 times per week to 3 or more times per day)

- 1-2 times/week (1)
- 3-4 times/week (2)
- 4-5 times/week (3)
- 6-7 times/week (4)
- 1-2 times/day (5)
- 3 or more times/day (6)

Q30 What is the most appealing benefit of increasing your fluid intake? Check all that apply.

- May prevent early uterine (Braxton Hicks) contractions (1)
- May prevent early labor (2)
- Feeling overall healthier (3)
- Reduced constipation (4)
- Reduced urinary tract infection risk (5)
- Reduced hemorrhoid risk (6)
- Help reducing fatigue (7)
- Help alleviate morning sickness (8)
- Help alleviate heartburn/indigestion (9)
- Help reducing headaches (10)
- Help reducing dizziness (11)
- Help reducing swelling (12)
- Help reduce overheating (13)
- Help body absorb essential nutrients into cells/transport vitamins, minerals, and hormones (14)
- Prevent low amniotic fluid levels (15)
- Weight maintenance (16)
- Better skin appearance/complexion (17)
- Improved physical performance (18)
- Improved cognitive performance (19)
- Other

Q31 If you selected “Other”, please explain.

Q32 Do you own a smartphone or tablet computer?

- Yes (1)
- No (2)
- Unsure (3)

Skip To: Q35 If Do you own a smartphone or tablet computer? = No

Q33 Have you ever installed an app on your phone or tablet to help you increase fluid consumption (hydration)?

- Yes (1)
- No (2)
- Unsure (3)

Q34 Have you ever owned a connected water bottle that tracks or provides reminders about fluid consumption? This water bottle connects to your phone via an app and tracks how much you drink throughout the day based on the weight (volume) of the water/fluid.

- Yes (1)
- No (2)
- Unsure (3)

Q35 How interested would you be in using a new smartphone application or device to aid in meeting the fluid consumption recommendations of 81 ounces per day?

- Yes (1)
- No (2)
- Unsure (3)

Q36 How likely is a smartphone application or device to help you meet the fluid consumption recommendations of 81 ounces per day?

- Yes (1)
- No (2)
- Unsure (3)

Q37 Which of the following smartphone or computer tools would be useful to help you comply with behavioral recommendations to increase fluid consumption (hydration; at least 80 ounces/day)?

| Educational materials (1) | - Useful (1) - Somewhat Useful (2) - Neither Useful or Not Useful (3) - Somewhat Not Useful (4) - Not Useful (5) |
| --- | --- |
| Scheduler with prompts/reminders (2) | - Useful (1) - Somewhat Useful (2) - Neither Useful or Not Useful (3) - Somewhat Not Useful (4) - Not Useful (5) |
| Self-monitoring tools for tracking behavior (3) | - Useful (1) - Somewhat Useful (2) - Neither Useful or Not Useful (3) - Somewhat Not Useful (4) - Not Useful (5) |
| Social media connections for support (4) | - Useful (1) - Somewhat Useful (2) - Neither Useful or Not Useful (3) - Somewhat Not Useful (4) - Not Useful (5) |
| Text messages with prompts/reminders/encouragement (5) | - Useful (1) - Somewhat Useful (2) - Neither Useful or Not Useful (3) - Somewhat Not Useful (4) - Not Useful (5) |
| Telephone coaching (6) | - Useful (1) - Somewhat Useful (2) - Neither Useful or Not Useful (3) - Somewhat Not Useful (4) - Not Useful (5) |
| Connected water bottle to measure/monitor volume of fluids consumed (7)  This water bottle will be connected to your phone via an app to track your water consumption throughout the day | - Useful (1) - Somewhat Useful (2) - Neither Useful or Not Useful (3) - Somewhat Not Useful (4) - Not Useful (5) |
| Automated notification triggered by technology that detected a lapse in preventive behavior (e.g., not drinking enough, eating poorly) (8) | - Useful (1) - Somewhat Useful (2) - Neither Useful or Not Useful (3) - Somewhat Not Useful (4) - Not Useful (5) |
| Call from clinic staff triggered by technology that detected a lapse in preventive behavior (e.g., not drinking enough, eating poorly) (9) | - Useful (1) - Somewhat Useful (2) - Neither Useful or Not Useful (3) - Somewhat Not Useful (4) - Not Useful (5) |
| Online community/forum for patients to share stories, advice, etc. (10) | - Useful (1) - Somewhat Useful (2) - Neither Useful or Not Useful (3) - Somewhat Not Useful (4) - Not Useful (5) |

Q38 How likely would you be to use a smartphone application or device on a regular basis to help you comply with behavioral recommendations to increase fluid consumption (hydration; 80 ounces per day)?

- Extremely Unlikely (1)
- Somewhat Unlikely (2)
- Neither Likely or Unlikely (3)
- Likely (4)
- Extremely Likely (5)
