# Supplementary material for: Every sip counts: Understanding hydration behaviors and user-acceptability of digital tools to promote adequate intake during early and late pregnancy
Source: PLOS Digit Health. 2024 May 7;3(5):e0000499. doi: 10.1371/journal.pdig.0000499 (PMC11075850; doi:10.1371/journal.pdig.0000499)
Supplement: Attachment — Submitted filename: Comments to PDIG-D-23-00237.docx [file pdig.0000499.s002.docx]

Comments to PDIG-D-23-00237

Line 87: Is it in the guidelines that pregnant women are encouraged to “track their water intake”? Please show me a reference.

Line 88: “New mothers” – do you mean first time being pregnant? Or first time giving birth? Please show me a reference for the statement on greater risk for maternal and infant mortality when not hydrating enough.

Line 94: I don’t understand what is written in the parathesis: N=300, 42% - is 42% 300 people, or is it 42% of the 300 people? The same for the next line on 14%

Line 99: Please use L or ounces throughout the text. I prefer L.

Line 106: 81 ounces – see previous comment

Line 107: MyFitnessPal app – I need a reference, or a more explanation for what this is and where it was produced.

Line 117: Is it possible to have a picture of a “smart water bottle”? I have never seen one.

Line 123: What is a “Just-in-time adaptive intervention”? Could you please elaborate?

Line 129: I do not understand why anyone want a bottle that overflows when the consumer has consumed enough if you want to increase water intake? If I had a bottle like that, I would just stop drinking to avoid the overflow??

Line 148-150: Why do women in later pregnancy have a decreased knowledge of hydration recommendation?

Line 153: You recruited for more than 1 year. Why did you only include 137? It seems like you have tried to reach out in many ways, but why did you end up with such small and monotonous sample size?

Line 163: What if you want to choose exactly 81 ounces?

Line 168: Do the pregnant women who answers this survey know what “food with high water content” is? Are there mentioned examples in the survey?

Line 199: Why was the survey for 6 months? So the women could change their answers? Or? Why was this needed?

Line 202: Why did so few complete the survey? Was it very long? Who was it difficult to complete for? How did they differ from those who was included?

Line 211: 8ounces or 81ounces?

Line 216: Is “preferred choice of beverage” referred to want they drink the most or what the like the most? In my opinion it could be misunderstood?

Line 223: The sentence ends with “and” – what did you want to say more?

Figure 1a to Figure 1d (Line 224 and forth): What is the purpose of asking the same question in 4 different ways?

Line 273: If you want to state that “pregnant women may benefit from useful strategies to increase knowledge…”, I think, you need to show that those who did track their fluid intake, had a higher fluid intake than those who did not.

Line 276: So you did investigate for a pattern in the answers? Those who did not meet hydration recommendation where they also those who did not know the hydration recommendation and did not track their fluid intake and so forth? Or? Please state this in the method section and mention in the result section.

Line 281: I do not see how your results “suggest there is a gap between women’s knowledge and their actual hydration behaviors” if the sentence before should be the reason for this statement. In the sentence before you present that pregnant women do not feel thirsty or forgot to drink and also that they drink water throughout the day – nothing about knowledge??

Line 284-285: Please put in a reference

Line 289: Fitbit – please give a reference (company, country, year)

Line 302: “wrist-worn sensors” – why did you not include this in the survey if you know it exists?

Line 321: Nice discussion of limitations of the study!

Additional: Did you compare answers of the survey and patient characteristics? Did you find any pattern when examining this? Maybe this could improve the planning of future studies within this field?
