# Supplementary material for: Every sip counts: Understanding hydration behaviors and user-acceptability of digital tools to promote adequate intake during early and late pregnancy
Source: PLOS Digit Health. 2024 May 7;3(5):e0000499. doi: 10.1371/journal.pdig.0000499 (PMC11075850; doi:10.1371/journal.pdig.0000499)
Supplement: Attachment — Submitted filename: Author Reply to Reviewer Comments 15September2023.docx [file pdig.0000499.s003.docx]

**PLOS Digital Health PDIG-D-23-00237
Every Sip Counts: Understanding hydration behaviors and user-acceptability of digital tools to promote adequate intake during pregnancy**

**Author Reply to Comments**

Thank you for the opportunity to revise and resubmit our manuscript to PLOS Digital Health. We have outlined each reviewer comment and our author reply below. In the manuscript, we have noted major changes in **red bold font** within the body of the manuscript.

| **Editor/Reviewer Comment** | **Author Response** | **Manuscript Changes** |
| --- | --- | --- |
| **Reviewer 1:** | | |
| For this journal, it is not allowed to have only one person as the corresponding author for your data. In the future, if you are not allowed to share your data in a de-identified fashion, you should put the ethics committee that oversees data sharing. Please refer to the guidelines for PLoS Digital Health here that might be helpful for future submissions. You can then update your data availability statement with the committee that oversees data sharing up at University Park. | Thank you. We will update this accordingly. | **--** |
| Also, in the introduction, you cited a Chinese study, but the majority of your respondents are white. You might want to put that article in the discussion section if you are aiming to broaden your reach for future research. | Completely understand your point. It was pretty difficult to find studies with methods similar to ours. This area of research is very limited. | **--** |
| Do not use contractions unless you are capturing responses from your participants, as it is considered not scholarly. | We will make changes to contractions. | Made changes throughout |
| This article is probably a better fit for International Journal of Behavioral Nutrition and Physical Activity or Journal of Nutrition. | Thank you for that suggestion. Since we received a revise and resubmit from PLOS Digital Health, we will resubmit and move forward to one of those journals, if rejected. | -- |
| **Reviewer 2** | | |
| Line 87: Is it in the guidelines that pregnant women are encouraged to “track their water intake”? Please show me a reference. | The guidelines don’t explicitly encourage pregnant women to track their intake but it is assumed that they should know how much they are drinking by setting a recommendation. | -- |
| Line 94: I don’t understand what is written in the parathesis: N=300, 42% - is 42% 300 people, or is it 42% of the 300 people? The same for the next line on 14% | Thank you for pointing that out. The authors can see how that is confusing and have updated the wording. | Lines 97-100 |
| Line 99: Please use L or ounces throughout the text. I prefer L.  Line 106: 81 ounces – see previous comment | Ounces have been changed to L throughout the manuscript. | Throughout the manuscript |
| Line 107: MyFitnessPal app – I need a reference, or a more explanation for what this is and where it was produced. | MyFitnessPal is a smartphone app that users can track their food and beverage intake, and exercise behaviors. | Added more details on lines 115-116 |
| Line 123: What is a “Just-in-time adaptive intervention”? Could you please elaborate? | Thank you for that question and pointing out the lack of detail. A Just In Time adaptive intervention is an intervention design that provides the right amount/type of support, at the best time for the participant based on their changing needs. | Added explanation to lines 128-129 |
| Line 129: I do not understand why anyone want a bottle that overflows when the consumer has consumed enough if you want to increase water intake? If I had a bottle like that, I would just stop drinking to avoid the overflow?? | We thought this exact same thing! We completely agree and think it is a weird idea for a water bottle! | -- |
| Line 148-150: Why do women in later pregnancy have a decreased knowledge of hydration recommendation? | Based on findings from Rosinger AY, et al., Variation in urine osmolality throughout pregnancy: a longitudinal, randomized-control trial among women with overweight and obesity. Eur J Nutr. 2022 Feb;61(1):127-140. doi: 10.1007/s00394-021-02616-x. Epub 2021 Jul 3. PMID: 34218315; PMCID: PMC8720908  it is suggested/assume that knowledge OR willingness to meet recommendations is decreased. However, these are exploratory findings and need to be further studied. | -- |
| Line 153: You recruited for more than 1 year. Why did you only include 137? It seems like you have tried to reach out in many ways, but why did you end up with such small and monotonous sample size? | Recruiting a sample of pregnant women has been a challenge, even if it is a simple survey. We had to modify our recruitment strategies to reach a target N. We also included questions in surveys that are included in other studies but not the entire questionnaire, so we wanted to use full data and not have mis-matched Ns. | -- |
| Line 163: What if you want to choose exactly 81 ounces? | They can actually choose whatever amount they want but we want to start them at 81 ounces as a goal. | -- |
| Line 168: Do the pregnant women who answers this survey know what “food with high water content” is? Are there mentioned examples in the survey? | Yes, there were examples of foods with high water content included in the question stem. All survey questions can be found in the Appendix. | Please see Appendix for questionnaire |
| Line 199: Why was the survey for 6 months? So the women could change their answers? Or? Why was this needed? | There was no reason behind the 6 month time frame. We didn’t want to keep it open too long. We probably could have left it open for longer but we wanted to get the data analyzed and use the data for pilot data in grant submissions.  Women were not able to access their survey once they submitted it. | -- |
| Line 202: Why did so few complete the survey? Was it very long? Who was it difficult to complete for? How did they differ from those who was included? | Recruiting a sample of pregnant women has been a challenge, even if it is a simple survey. We had to modify our recruitment strategies to reach a target N. We also included questions in surveys that are included in other studies but not the entire questionnaire, so we wanted to use full data and not have mis-matched Ns. | -- |
| Line 211: 8ounces or 81ounces? | Thank you for noticing this typo. Has been change. | Line 218 |
| Line 216: Is “preferred choice of beverage” referred to want they drink the most or what the like the most? In my opinion it could be misunderstood? | This question referred to which beverage they drink the most. We completely understand the misunderstanding and will revise the question in future studies. | -- |
| Line 223: The sentence ends with “and” – what did you want to say more? | Thank you for pointing this out. This has been removed. | Line 235 |
| Figure 1a to Figure 1d (Line 224 and forth): What is the purpose of asking the same question in 4 different ways? | These questions are very similar but are getting at different behaviors – without thinking vs remembering to drink are pretty different. We completely understand how closely related they are but the slight differences can tell us a good amount about their conscious and unconscious behaviors. | -- |
| Line 273: If you want to state that “pregnant women may benefit from useful strategies to increase knowledge…”, I think, you need to show that those who did track their fluid intake, had a higher fluid intake than those who did not. | Thank you for this point. The sentence has been updated to reflect the refocus of the findings. We do aim to do these analyses in the future! | Line 285 |
| Line 276: So you did investigate for a pattern in the answers? Those who did not meet hydration recommendation where they also those who did not know the hydration recommendation and did not track their fluid intake and so forth? Or? Please state this in the method section and mention in the result section. | We did not. We definitely will go this direction with this data and in future studies. We really were focused on the larger picture of the data but will definitely be exploring more specific characteristics that may influence water intake and help build stronger studies/methods. Very much appreciate the suggestion. |  |
| Line 281: I do not see how your results “suggest there is a gap between women’s knowledge and their actual hydration behaviors” if the sentence before should be the reason for this statement. In the sentence before you present that pregnant women do not feel thirsty or forgot to drink and also that they drink water throughout the day – nothing about knowledge?? | Added a sentence for clarity and to connect the results and discussion points. | Line 297 |
| Line 284-285: Please put in a reference | Updated the wording to reflect we were discussing the current findings, not those from another study. | Lines 291-293 |
| Line 289: Fitbit – please give a reference (company, country, year) | That is included in the manuscript – added country to the citation | Lines 302-304 |
| Line 302: “wrist-worn sensors” – why did you not include this in the survey if you know it exists? | We weren’t incredibly focused on the wrist-worn sensors mostly because we wanted to focus on the app/water bottle pair. These are something that we are going to explore in the future. | -- |
| Line 321: Nice discussion of limitations of the study! | Thank you! Greatly appreciated! | -- |
| Did you compare answers of the survey and patient characteristics? Did you find any pattern when examining this? Maybe this could improve the planning of future studies within this field? | Similar to the comment above, we did not get into the more specific/granular patterns/comparisons to characteristics for this study. We will definitely be conducting these analyses to strengthen our future studies and methods. Greatly appreciate that suggestion! | -- |
| **Reviewer 3** | | |
| Introduction: The author emphasizes (L147-150) on the difference in knowledge of hydration recommendations and willingness of using digital tool according to the age of pregnancy. However, this is not reflected in the title. | Thank you for noticing this important point. We have updated the title to reflect the age of pregnancy. | Line 5 |
| L153: To avoid a possible confusion, I was wondering if it is not wise to fully write OB/GYN even if it is known what it stands for. Why flyers were not also posted in antenatal care facilities where majority of pregnant women frequently visit in? I assume that OB/GYN clinics are rarely visited and especially for a serious issue or a referral case. | We have written out obstetrics and gynecology for clarity. “Community sites” include various locations around the local area – which included antenatal care facilities but wanted to specify we were targeting currently pregnant women, who visit their OB regularly. In our location, most women did visit their OB regularly but also have access to flyers and social medial posts. | Line 159 |
| L154: It not clearly shown how participant were screened against eligibility criteria. I would also like to know if an interested participant had to request for the link to access the online survey. | We have updated the screening procedures and women could request the link to access the online survey but the study staff would screen them to determine eligibility, | Lines 160-163 |
| The study setting is not defined. Was the study conducted in a particular community? | The study was advertised across the United States – there wasn’t one particular community that was targeted. | Line 160 |
| L156-157: I think that a participating woman was not aware of the $50 gift card before otherwise it could be a source of bias. | We understand this but many women will not participate if they do not have a chance of receiving some sort of compensation for their efforts. | -- |
| I think it would be good idea to ask question about types of apps used to track water intake because different apps may have different effect on participants acceptability; some may be more acceptable than others. | We agree and that is something we are going to explore further. This survey was done to get an initial sense of if women would even be interested in using an app. | -- |
| Nothing is mentioned concerning informing to participants about the study and getting their consent. | All women were consented into the study. The link that included the survey, also included the consent form. | Line 166 |
| Why was the survey remained opened for exactly 6 months? any particular reason/explanation for that? | There was no reason behind the 6 month time frame. We didn’t want to keep it open too long. We probably could have left it open for longer but we wanted to get the data analyzed and use the data for pilot data in grant submissions. | -- |
| I would be better to state how data were managed for analysis to ease the reader understanding. | Thank you for this suggestion. We have added more detail in the methods. | Lines 206-207 |
| Table 1 describing participant demographics of pregnant women shows that two women were retired and I doubt that a woman can be pregnant when she has reached an age of retirement… | We thought that was interesting as well. However, these women could have been in the military or disabled and selected retired. We did not have an option for disabled but we will add that in the future. | -- |
| L211: a typo “8ounces” | Thank you for noticing this typo. Has been change. | Line 218 |
| Table two show that only 7 (5%) participants were using a tracking app what seems to be in contrast with what is reported concerning interest in use of new app (L247-252) | Yes, we found this interesting as well. However, just because someone isn’t using an app, doesn’t limit their interest in using an app in the future but still a very interest difference. | -- |
| L271: I hope it is an assumption that most women own a smartphone or a tablet/computer. I have not seen it in the results | We did ask this question and 98% of the women responded that they owned a smartphone or tablet/computer. We did not think this was meaningful to include in the results, so we essentially are assuming this. | -- |
| I recommend the author to search more other evidences to discuss his findings on the two aspects; knowledge and use of digital tools in tracking water intake. | Greatly appreciate this suggestion. However, after much searching, it was very difficult to find other studies/evidence currently published in this area. This is why we conduc | -- |
| The result section is heavy reporting much data. The author should report key findings to guide the conclusion | Thank you for this suggestion. We have already reduced the results to what is included here. We found these were the main results that helped build our story and conclusions. | -- |
| **Journal/Editor Comments** |  |  |
| Please send a completed 'Competing Interests' statement, including any COIs declared by your co-authors. If you have no competing interests to declare, please state "The authors have declared that no competing interests exist". Otherwise please declare all competing interests beginning with twhe statement "I have read the journal's policy and the authors of this manuscript have the following competing interests:" | Authors do not have any competing interests | Line 44-45 |
| Please amend your detailed Financial Disclosure statement. This is published with the article. It must therefore be completed in full sentences and contain the exact wording you wish to be published.  a. State the initials, alongside each funding source, of each author to receive each grant. b. If any authors received a salary from any of your funders, please state which authors and which funders.  If you did not receive any funding for this study, please simply state: “The authors received no specific funding for this work.” | The authors did not receive direct funding from the funding sources listed. Penn State received funding for the REDCap data capture system that the study used to collect and store data.  None of the authors received a salary from the funder. | Noted in lines 360-361 |
| In the online submission form, you indicated that "The data can be accessed through contacting the corresponding author". All PLOS journals now require all data underlying the findings described in their manuscript to be freely available to other researchers, either 1. In a public repository, 2. Within the manuscript itself, or 3. Uploaded as supplementary information. This policy applies to all data except where public deposition would breach compliance with the protocol approved by your research ethics board. If your data cannot be made publicly available for ethical or legal reasons (e.g., public availability would compromise patient privacy), please explain your reasons by return email and your exemption request will be escalated to the editor for approval. Your exemption request will be handled independently and will not hold up the peer review process, but will need to be resolved should your manuscript be accepted for publication. One of the Editorial team will then be in touch if there are any issues. | We will provide the de-identified data. | -- |
| Please follow the journal instructions for manuscript preparation. I assume the abstract should be unstructured. | We have unstructured the abstract. | Abstract has been unstructured. |
| Please add appropriate keywords (preferably selected based on the MeSH terms) after the abstract. | MeSH terms added below the abstract. | Line 66 includes MeSH terms |
| Please use appropriate subheadings in your methods section (study design, research participants, research instrument, data collection and analysis, etc.) to organize and improve readability of this section | We made these subheading changes. | Changes made to Methods section headers |
| Please add the questionnaire as an Appendix. | The questionnaire has been added as an Appendix. | Added as appendix. |
| Please ensure that the data for all questions have been presented in the results section either via Table or Figure. | For all questions included in the methods, all data is provided in the results. | -- |
| References older than 10 years ago need to be updated. | We understand the need for the most updated/recent references but these references are some of the only publications in this research area. Also, there are references that are frequently used due to their long standing history in this field. | -- |
