# Supplementary material for: Every sip counts: Understanding hydration behaviors and user-acceptability of digital tools to promote adequate intake during early and late pregnancy
Source: PLOS Digit Health. 2024 May 7;3(5):e0000499. doi: 10.1371/journal.pdig.0000499 (PMC11075850; doi:10.1371/journal.pdig.0000499)
Supplement: Attachment — Submitted filename: Author Reply to Reviewer Comments 12February2024.docx [file pdig.0000499.s004.docx]

**PLOS Digital Health PDIG-D-23-00237
Every Sip Counts: Understanding hydration behaviors and user-acceptability of digital tools to promote adequate intake during early and late pregnancy**

**Author Reply to Comments**

Thank you for the opportunity to revise and resubmit our manuscript to PLOS Digital Health. We have outlined each reviewer comment and our author reply below. In the manuscript, we have noted major changes in **red bold font** within the body of the manuscript.

| **Editor/Reviewer Comment** | **Author Response** | **Manuscript Changes** |
| --- | --- | --- |
| **Reviewer 4** | | |
| Line 162, 163: The survey provided an open answer question “Do you know how much water you should be drinking in a day (in ounces?)”, and the answer was dichotomized into less than 81 ounces and greater than 81 ounces. While this serves as a proxy of the water consumption adequacy, the authors should justify why this cutoff was chosen, since recommended water intake is largely based on other metrics including age, BMI, , activity level, comorbidities..etc. The recommended water intake amount during pregnancy according to ACOG on their official site 8 to 12 cups (64 to 96 ounces) ([https://www.acog.org/womens-health/experts-and-stories/ask-acog/how-much-water-should-i-drink-during-pregnancy](https://nam10.safelinks.protection.outlook.com/?url=https%3A%2F%2Fwww.acog.org%2Fwomens-health%2Fexperts-and-stories%2Fask-acog%2Fhow-much-water-should-i-drink-during-pregnancy&data=05%7C02%7Camp34%40psu.edu%7C03c61bd3a6124c2ae5aa08dc22a6a36f%7C7cf48d453ddb4389a9c1c115526eb52e%7C0%7C0%7C638423346298555990%7CUnknown%7CTWFpbGZsb3d8eyJWIjoiMC4wLjAwMDAiLCJQIjoiV2luMzIiLCJBTiI6Ik1haWwiLCJXVCI6Mn0%3D%7C0%7C%7C%7C&sdata=Kjsxc8hoGFXHLZCZHCpJergyBNF6Xjasxeo08ZjSsE4%3D&reserved=0) ) | Thank you very much for this comment. According to the Institute of Medicine’s dietary reference manual for water, potassium, sodium, chloride, and sulfate, it is recommended that pregnant women obtain approximately 2.3L of their daily total water of from beverages, including water. Also, on page 1, lines 88-89, the authors state the recommendations for pregnant women but have also added the ACOG recommendations on page 1, lines 89-92 to better justify this in the revision. We have also addressed the use of the dichotomized variable in the limitations. | Please see lines 89-92 on page 4 (Introduction): Added a sentence explaining the ACOG guidelines and why there is a range provided instead of a set number for intake recommendations (reference 9).  **However, the American College of Obstetricians and Gynecologists recommend a range of 8 to 12 cups/day (64 to 96 ounces/day; 1.9 – 2.8 L/day) [9], which accounts for varied needs, due to greater body composition and body mass, living in hotter temperatures and engaging in physical activity [10,11].**  Please see lines 181-184 on page 7 (Methods, Study Design): Added a justification statement of using 2.4L based on the IOM’s dietary reference manual (reference 7).  **These dichotomized cut-offs were used based on the recommendations the Institute of Medicines Dietary Reference manual [7], which states that pregnant women ages 19-50 years old should obtain approximately 2.3 L of their total daily water intake from beverages, including drinking water [7].**  Please see lines 357-360 on page 14 (Strengths and Limitations): Added a limitation regarding the use of the dichotomized variable for recommended water intake.  **Lastly, there are varied ranges in the literature for recommended water intake values (1.9 – 2.8 L; 64 - 96 ounces) [9]. This study used a dichotomous cutoff of 2.3 L (81 ounces) [7] to classify meeting vs not meeting hydration recommendations for feasibility purposes but future work should explore variations in water intake as the literature suggests [7,9].** |
| The citations listed on line 106 regarding the recommended amount of water intake: meet the hydration guidelines of consuming at least 81 ounces of water from ~8-36 weeks’ gestation [15,16]. Both articles did not explicitly state “81 ounces” as the recommended amount of water intake, other citations for justifying the “81 ounce” should be provided. | Thank you for this comment. The authors have added the citation from prior research that we used for providing a justification for the cut-off used for classification of meeting vs not meeting the recommendations. | Please see lines 89-92 on page 4 (Introduction).  Added citation for sentence in lines 108-111; As part of a larger, multicomponent intervention, women in the intervention group were encouraged to track their water intake, increase intake of water-dense foods, and meet the hydration guidelines of consuming at least 2.4 L of water from ~8-36 weeks gestation [**7**,15,16]. |
| The completion rate of surveys was 34%. Could the authors specify the predominant channel of recruitment for those who completed the survey (patients who were included in the analysis)? Understanding the behavioral profile of participants sourced through diverse platforms such as social media, community flyers, OB/GYN clinics, and University websites could offer nuanced insights. | Thank you for this comment. There were multiple recruitment and data collection streams. Statements reporting recruitment rates via social media, internet, word of mouth, and flyer at provider’s offices and reasons for the low completion rate were added to the results section. | Please see lines 224-230, page 9: Added data regarding recruitment streams.  **The majority of women were recruited through social media (50.4%) and from across the United States. Outside of social media, 31.4% of women heard about the study from other internet sources (Google, Research Match), 12.4% heard about the study via word of mouth, and 8.8% saw a flyer at their provider’s office. The survey link was included on all social media posts allowing direct access and ability to complete the survey easily and the link was shared easily. A low completion rate could be due to starting the survey and no longer wanting to participate due to various factors such as lack of compensation, the length of the survey, and time constraints.** |
| The tables presented in the manuscript are mostly descriptive data. But regarding the statistics of comparing between early and late pregnancy, only 137 samples were analyzed between 2 groups (early vs late), yet the authors chose One-way ANOVA: a parametric test which is used for 3 or more groups for the analysis. It is unlikely that the numbers from the small sample size would be normally distributed. I recommend re-doing the analysis with a non-parametric test for 2 groups (Wilcoxon rank sum test). Moreover, considering the binary nature of most survey items (yes/no), shouldn't the chi-squared test be apt for comparing categorical variables across groups? The omission of this in the methodology is confusing. | Thank you for this comment.  The authors have revised the analyses and re-ran group comparisons using the Mann-Whitney U test. The results section has been updated to reflect the change in analyses.  For the binary questions, the authors revised the analysis plan and re-ran the analyses as chi-squared tests. Results from the chi-squared tests were the same and there were no significant differences between the groups. | Please see lines 216, page 8: Updated the Data Collection and Analysis section to include that Mann-Whitney U and chi-squared tests were used to determine group differences.  Please see lines 240-241, page 10 for the updated results for the Mann-Whitney U test.  **Women in early pregnancy reported significantly lower recommended values of water/day (1.5 L) compared to women in late pregnancy (2.4 L; U = 666.5, n1 = 15, n2 = 60; p = 0.004).** |
| **Reviewer 5** | | |
| Some minor errors still need to be corrected: lack of close parenthesis in line 82, and in-text citation formatting in lines 291 and 292. Please follow PLOS guidelines for the citation format. | Thank you for catching these issues. These have been corrected. | 1. Closed the parenthesis on line 85.  2. Have updated the in-text citation and reference list to adhere to PLOS guidelines – updated in-text citation numbers and references have been updated (updates are in bold red throughout the manuscript and in the reference list; page 16). |
